# Multiple Congenital Epulis of the Mandibular Ridge: A Case Report

**Published:** 2012

**Authors:** Mehran Hiradfar, Nona Zabolinejad, Mohammad Gharavi, Sadaf Sebt

**Affiliations:** 1 *Department of Pediatric Surgery, Mashhad University of Medical Sciences, Mashhad, Iran.*; 2 *Department of Pathology, Mashhad University of Medical Sciences, Mashhad, Iran.*; 3 *Department of Anesthesiology, Mashhad University of Medical Sciences, Mashhad, Iran.*; 4 *Medical Student, Islamic Azad University of Mashhad, Iran.*

**Keywords:** Congenital epulis, Congenital gingival granular cell tumor, Mandible

## Abstract

Congenital epulis is a very rare benign soft-tissue tumor of uncertain histogenesis, which is also known as “gingival granular cell tumor of the newborn”. It occurs almost exclusively as a single tumor along the alveolar ridge of the maxilla in newborn females. Although congenital epulis is strikingly similar to the more common adult granular cell tumor histologically, in contrast to the latter congenital epulis cells are negative for S-100 protein. This case report describes a 15-day-old female infant with multiple congenital epulis of the mandibular alveolar ridge.

## Introduction

Congenital epulis is a rare benign soft-tissue tumor of uncertain histogenesis, which is also known as “gingival granular cell tumor of the new-born”. The first description of this lesion is attributed to Neumann in 1871(1). The Greek word epulis means “swelling on the gingiva”. The tumors occur almost exclusively along the alveolar ridge of the maxilla in white female newborns and are not associated with congenital malformations (except one documented case of association with a genital anomaly) or deformities of the teeth. However, when large or multiple they may cause respiratory or feeding problems (2-4).

In this report, we describe the case of a 15-day-old female infant with multiple congenital epulis on her mandibular alveolar ridge and describe the clinical, pathological and differential diagnosis. 

## Case Report

The patient was a 15-day-old female infant born at 39 weeks gestation weighing 3200 g. She was born by normal vaginal delivery and had Apgar scores 9/10 at both 1 and 5 minutes. She was referred to our pediatric surgery department due to the presence of large intra-oral cavity masses that were protruding out of the oral cavity and causing feeding problems. These masses, first noticed at birth, originated from the anterior mandibular alveolus, occupied the entrance of oral cavity and partially protruded out of it.

**Fig 1:**
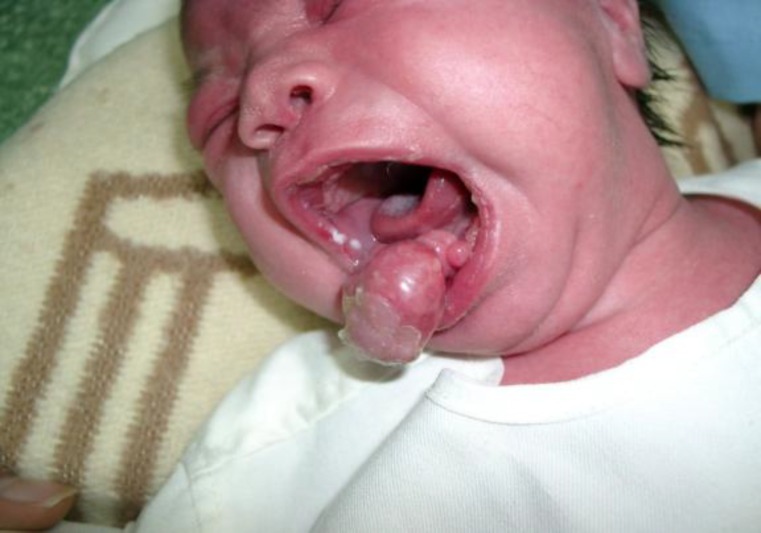
A large pedunculated mass and two other smaller tumors on the mandibular alveolar ridge in a neonate female

Intra-oral examination showed a reddish, pedunculated soft tissue mass exhibiting a smooth erythematous surface with some scales on the part outside the oral cavity. The mass measured 5 x 4 cm and was attached to the anterior gingiva of the mandible. Another two similar, smaller tumors were observed near the larger mass (Fig. 1). On palpation they were firm, not compressible and non-tender. The masses prevented normal closure of the mouth but posed no immediate airway concerns. Feeding by a nasogastric tube was instituted. General physical examination and other laboratory tests were otherwise normal.

**Fig 2:**
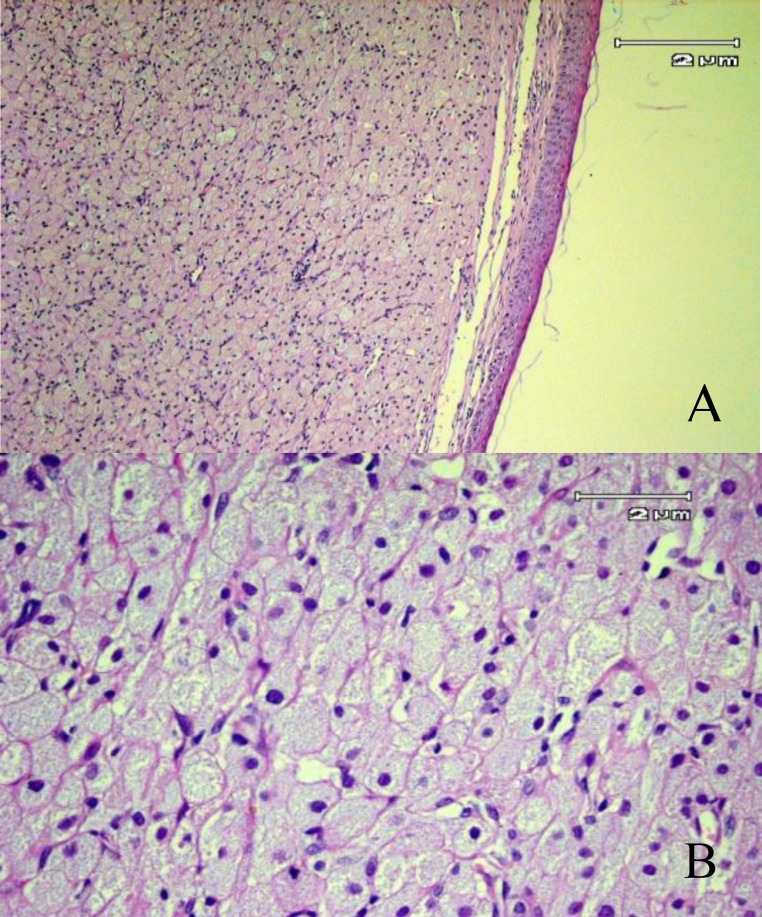
**(A)** Large polygonal cells with abundant granular cytoplasm that are covered with flattened squamous stratified epithelium (H&E×40). (**B**) The same specimen as in (A) at a higher magnification (H&E×400)

The lesion was completely excised at the base with bipolar cautery under general anesthesia, with minimal intraoperative hemorrhage. Histologic examination of the specimens revealed well circumscribed lesions covered with squamous epithelium. They were composed of homogeneous polygonal cells with granular eosinophilic cytoplasm and centrally located basophilic nuclei in a vascularized stroma (Fig. 2).

The cytoplasmic granules were PAS **(Periodic acid-Schiff ) **positive. Immunohistochemically, the tumor cells were diffusely positive for vimentin, and negative for S-100 protein, actin and desmin. These findings were consistent with congenital epulis. No recurrence occurred during 3 years of follow up.

## Discussion

Congenital epulis is a rare benign lesion of uncertain histogenesis. Although this tumor looks histologically like an acquired granular cell tumor (also called granular cell myoblastoma or Abrikossoff’s tumor), it has its own characteristics. Congenital epulis is a reactive or degenerative lesion with a mesenchymal origin rather than a true neoplasm (5). Recent studies favor myofibroblasts as the cell of origin (5,6). Congenital epulis usually present at birth as an incidental finding; however, when large or multiple, they may precipitate respiratory or feeding problems (2, 3). The lesions occur sporadically, and no familial tendencies have been described (2).

Dash and colleagues reported on one of the largest series of congenital epulis to date, for which the authors collected data from 50 cases. Congenital epulis has an 8:1 incidence in females and a 3:1 incidence of occurring at maxillary alveolar sites (7). Considering the high incidence in females; an endogenous (intrauterine) hormonal stimulus has been proposed but was disproven by the absence of receptors for estrogen and progesterone (3). Congenital epulis clinically appears as a pedunculated protuberant mass. The size of the epulis varies from a few mm to 9 cm (5). Our case was a female infant, in whom the lesion occurred in the anterior mandibular gum pad. Congenital epulis is usually a single lesion, but in 10% of cases it may be multiple, as in our case (2,3,5).

Histologically, congenital epulis is strikingly similar to the more common adult granular cell tumor. They are both composed of large round cells with abundant granular cytoplasm and small eccentric nuclei with occasional small nucleoli. There is a prominent vascular stroma with perivascular lymphocytes and histiocytes. Entrapped non-neoplastic odontogenic epithelium may be present in some cases. In contrast to granular cell tumors, there is no pseudoepitheliomatous hyperplasia of the overlying squamous mucosa in congenital epulis, and no nerve bundles are seen within this lesion. Furthermore, congenital epulis was shown to be consistently negative when immunostained with S-100 protein, in contrast to adult granular cell tumors, which are derived from Schwann cells (2,4,5). Similarly, the tissue removed from our patient was negative for S-100 protein. 

Congenital epulis is often misdiagnosed before surgery because of its rarity and a lack of awareness of the condition by clinicians. The differential diagnosis of a large mass in the fetal or neonatal oral cavity includes hemangioma, lymphatic malformations, teratoma, pigmented neurectodermal tumor of infancy, rhabdomyoma and rhadomyosarcoma (2,3,6). 3D ultrasound and magnetic resonance imaging examinations may show the lesion in the last weeks of pregnancy, although the findings are not specific (8).

Complete surgical excision is the treatment of choice but surgery should not be radical to minimize the danger of damaging underlying alveolar bone and developing tooth buds (9). There are also some case reports that have documented spontaneous regression, especially in very small lesions. In this situation, regular monitoring of the lesion for regression has been advocated as an acceptable clinical approach (10). No recurrence has been reported even after incomplete excision (2,5,9).

## Conclusion

Congenital epulis is a rare benign soft-tissue tumor of uncertain histogenesis. Cases of spontaneous regression, lack of recurrence even after incomplete resection and lack of malignant transformation suggest that congenital epulis is a non-neoplastic lesion and needs to be differentiated from an acquired granular cell tumor.
